# MicroRNA-143 expression inhibits the growth and the invasion of osteosarcoma

**DOI:** 10.1186/s13018-022-03127-z

**Published:** 2022-04-13

**Authors:** Pei Zhang, Jiale Zhang, Huahong Quan, Jingcheng Wang, Yuan Liang

**Affiliations:** 1 Department of Orthopedics, The Second Xiangya Hospital of Central South University, Changsha, 410011 Hunan China; 2 Department of Orthopedics, Clinical Medical College, Yangzhou University, Northern Jiangsu People’s Hospital, Yangzhou, China; 3 Department of Graduate, Dalian Medical University, Dalian, 116044 Liaoning China

**Keywords:** MicroRNA-143, Osteosarcoma, Prognosis

## Abstract

**Background:**

Osteosarcoma (OS) is a common malignant tumor, which occurs in the metaphysis of the long diaphysis from mesenchymal tissue. Previous studies have indicated that expression of microRNA-143 (miR-143) could affect cancer cell proliferation, migration and invasion. The present research was performed to figure out whethermiR-143 expression inhibits the growth and the invasion of OS.

**Methods:**

We conducted a literature search in the electronic databases of Medline, Embase, Web of Science, and the Cochrane Library, SinoMed, WanFang, China national knowledge infrastructure (CNKI) until January 2022. We used Review Manager 5.3 software to conduct our research.

**Results:**

Twelve eligible articles were included, 5 articles were reported outcomes about mice, 11 articles were reported outcomes about human. The results of mice demonstrated that the miR-143 group had significantly better results in tumor volume, tumor weight and survival rate. The results of human demonstrated that the high level of miR-143 group had significantly better results in the 3-year, 4-year, and 5-year survival rate, lung metastasis and tumor grade.

**Conclusions:**

MiR-143 has potentially important value in the treatment and prognosis of OS. However, more reliable animal and clinical trials are needed before miR-143 based therapies can be transferred from animal studies to human applications.

## Introduction

OS is a common malignant tumor, occurs in the metaphysis of the long diaphysis from mesenchymal tissue [1–3]. This bone malignancy mainly affects the young people and adolescents. Despite the fact that recent advances in treatments that surgical-wide resection combine with chemotherapy and radiotherapy treatments, approximately 40–50% of the patients occur pulmonary metastasis [4, 5]. Among these patients, the 5-year survival rate is just 28%. Thus, it is important to develop a novel therapeutic approach in OS treatment.

MiRNA with a length about 18–26 nucleotides, is a class of endogenously expressed and non-coding small RNA. Because one miRNA can target several mRNAs, they are vital components of a large regulatory network, miRNAs can regulate multiple cellular functions [6–8]. Previous studies have indicated that miRNAs are aberrantly expressed or mutated in the development of cancers [9, 10]. The mechanism of miRNA is helpful for the diagnosis and treatment of OS. Studies show that miR-143 has featured as tumor suppressors in various tumors, such as ovarian cancer, glioblastoma, pancreatic cancer, and so on [11–13]. It was also reported the expression level of miR-143 decreased in OS tissues and cells, and low expression level of miR-143 could accelerate cell proliferation, migration and invasion [6, 14–16]. MiR-143 may have potentially important value in the treatment and prognosis of OS. Therefore, we systematically collected all available literatures about miRNA-143 and OS in the mice models and human to discuss the function of miR-143.

## Methods

The Preferred Reporting Items for Systematic reviews and Meta-Analysis (PRISMA) guidelines were followed in this study. The research protocol for this review was determined by all coauthors before the literature searches begun, and the study protocol was published online at the PROSPERO (https://www.crd.york.ac.uk/prospero/) under registration number CRD42020191937.

### Inclusion and exclusion criteria

To be included in our analysis, the study had to follow inclusion criteria belowing: (1) Studies compared low and high miRNA-143 expression in mice or human with OS. (2) Outcomes were not limited to pool. (3) Studies published in English. Studies were excluded: (1) Duplicate publication. (2) Studies with insufficient data. (3) Studies not published in English. (4)Conference, case report, or cadaver studies.

### Literature search

A literature search was conducted to search studies compared low and high miRNA-143 expression in mice or human with OS. The searched terms were following: “osteosarcoma”, “microRNA-143”, “miRNA-143”. The Boolean operators and/or were used to combine them. Medline, Embase, Web of Science, and the Cochrane Library, SinoMed, WanFang, and CNKI were searched to retrieve related studies until January 2022.

### Data extraction

The following information was respectively extracted from each of the included studies by two investigators (ZJ and QH). The basic data of mice were extracted from the studies: author, year of publication, the number of animals, characteristics of animals, tumor volume, tumor weight, mice survival number.

The basic data of human were extracted from the studies: author, country, year of publication, the number of patients, gender, age, anatomical site, metastasis, tumor size, tumor grade, survival curve, expression level of miR-143, When disagreement existed, it was resolved by consulting another investigator (LY).

### Quality assessment

The quality of included studies was respectively assessed by two investigators (ZJ and QH). The quality of the animal studies was assessed according to CAMARADES [17]. Risk of selection, performance, detection, attrition and reporting bias was assessed using SYRCLE’s risk of bias tool [18]. The quality of the human studies was assessed according to the Downs and Black [16] and the Newcastle–Ottawa Scale (NOS) [19] quality assessment methods. A total NOS score was 9* and if the NOS score was over 6*, it would be regarded as higher quality research. A higher score was recognized as better quality research. Any different opinions were resolved by a third reviewer (WJ).

### Statistical and sensitivity analysis

The meta-analysis was conducted using Review Manager 5.3. For continuous outcomes, a standard mean difference (SMD) and 95% confidence interval (CI) were used. For dichotomous data, the risk difference (RD) with 95% CI was calculated as the summary statistics. Statistical heterogeneity was assessed using the value of *P* and *I*^2^. If *P* was > 0.1 and *I*^2^ was < 50%, the fixed-effects model was used; otherwise, the random-effects model was used to do analysis. Random-effects models were used to reduce heterogeneity. Data analysis was carried out by using Review Manager 5.3. Sensitivity analysis was performed to assess the accuracy of our results through the exclusion of eligible studies once time.

#### Data collection and data processing

The miR-143 related data-sets in GEO (http://www.ncbi.nlm.nih.gov/geo) were used to make analysis. Background correction and normalize were processed by the R package ‘affy’ from the Bioconductor project. The DE-miRNAs analysis was conducted by the R package ‘limma’ (Filter: |log2FC|> 1; *P* < 0.05).

## Results

### Study selection

**Fig. 1:**
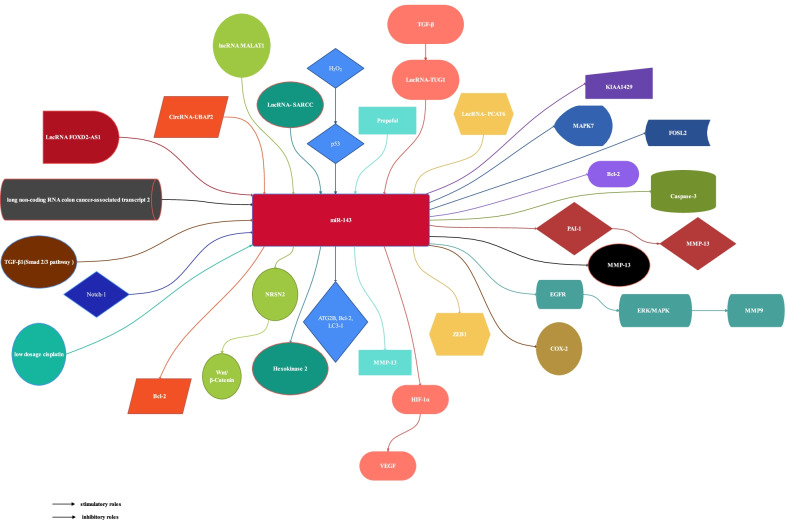
The role of miR-143 in OS

**Fig. 2:**
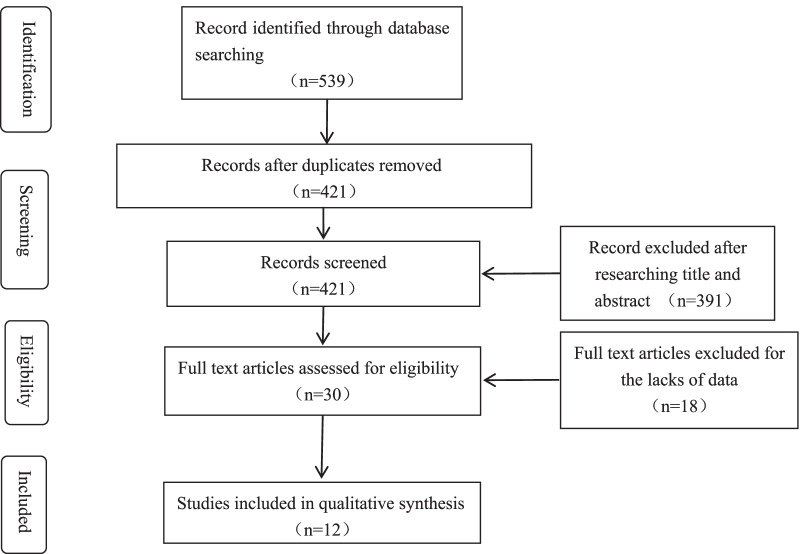
Flow chart of the study selection procedure

After the initial scanning of the titles and abstracts, 4 studies related to other sarcoma were excluded [20–23], 30 studies [1, 3–8, 14, 15, 24–44] related to OS that met the inclusion criteria were reviewed for full-text screening. After full texts assessed for eligibility, 18 studies were excluded due to lack of data. Finally, 12 eligible articles [1, 4, 7, 15, 25, 28–30, 33, 37, 38, 40] were included, 5 studies reported the outcomes about mice [7, 28, 30, 37, 38], 11 studies reported the outcomes about human [1, 4, 7, 15, 25, 28–30, 33, 37, 40]. The information of 29 studies [1, 3–8, 14, 15, 24–39, 41–44] related to OS were shown in Table 1 and the information of 4 studies [20–23] related to other sarcoma were showed in Table 2. The characteristics of the studies about mice in the meta-analysis were showed in Table 3 and the characteristics of the studies about human in the meta-analysis were showed in Table 4.The role of miR-143 in OS was shown in Fig. 1.The selection process was shown in Fig. 2.

**Table 1:** The information of OS

References	Genes/proteins affected	Functions
Zhang [41]	LncRNA FOXD2-AS1┤ miR-143	LncRNA FOXD2-AS1 knockdown inhibits the resistance of human osteosarcoma cells to cisplatin by inhibiting miR-143 expression
Li [42]	lncRNA MALAT1/miR-143/NRSN2/Wnt/β-Catenin	Bone marrow mesenchymal stem cells-derived extracellular vesicles promote proliferation, invasion and migration of osteosarcoma cells via the lncRNA MALAT1/miR-143/NRSN2/Wnt/β-Catenin Axis
Bi [43]	Long non-coding RNA colon cancer-associated transcript 2┤ miR-143	Long non-coding RNA colon cancer-associated transcript 2 knockdown proliferation and metastasis of osteosarcoma cells by inhibiting miR-143 expression
Yang [44]	–	MiR-143-3p function as diagnostic and prognostic markers for osteosarcoma
Han [14]	miR-143-3p ┤KIAA1429	Knockdown of KIAA1429 or ectopic overexpression of miR143-3p could repress stemness cell properties and the inhibition could be partly abolished by overexpression of KIAA1429
Wu [39]	LncRNA- PCAT6┤miR-143-3p /ZEB1	LncRNA-PCAT6 Aggravates Osteosarcoma Tumorigenesis via the MiR-143-3p/ZEB1 Axis
Wen [1]	LncRNA-SARCC → miR-143┤ Hexokinase 2	LncRNA-SARCC sensitizes OS to cisplatin through the miR-143-mediated glycolysis inhibition by targeting Hexokinase 2
Yu [5]	TGF-β → LncRNA-TUG1 ┤miR-143-5p┤HIF-1α → VEGF	Long non-coding RNA Taurine upregulated gene 1 promotes OS cell metastasis by mediating HIF-1α via miR143-5p
Jerez [24]	–	MiRNAs (miR-21-5p, miR-143-3p, miR-148a-3p and 181a-5p) present in EVs may regulate the metastatic potential of OS cell lines by potentially inhibiting a network of genes (MAPK1, NRAS, FRS2, PRCKE, BCL2 and QKI) involved in apoptosis and/or cell adhesion
Zhao [25]	–	The expression of miR-143 was significantly lower in low-grade OS compared to high-grade OS. The expression of miR-143 was significantly lower in OS with lung metastasis compared to OS without lung metastases
Hou [6]	miR-143-3p┤MAPK7	Mechanism of miR-143-3p inhibiting proliferation, migration and invasion of OS cells by targeting MAPK7.
Sun [7]	miR-143 ┤ FOSL2	MiR143-3p directly and negatively targets FOSL2 to affect OS characteristics
Dong [15]	miR-143-3p ┤MAPK7	MiR-143 suppress the proliferation, migration and invasion ability of OS cells via downregulating the MAPK7 expression
Zhang [26]	CircRNA-UBAP2 ┤miR-143┤Bcl-2	Circular-RNA UBAP2 was found to inhibit the expression of microRNA-143 (miR-143), thus enhancing the expression and function of anti-apoptotic Bcl-2
Li [27]	Low dosage cisplatin → miR-143	Under low dosage cisplatin treatment, miR-143 may be activated in induce the expression of Bcl-2, which further impede the cell proliferation
WH Li [28]	miR-143┤Bcl-2 miR-143 → Caspase-3	MiR-143 could inhibit Bcl-2 expression, causing Caspase-3 activation, thus inducing apoptosis in OS cells
Hirahata [4]	miR-143-3p ┤PAI-1 → MMP-13	PAI-1, a target gene of miR-143, regulates invasion and lung metastasis via enhancement of MMP-13 expression and secretion in human OS cells
Liu [29]	miR-143┤Bcl-2	mRNA and protein levels of Bcl-2 were depressed after over-expression of miR-143. Meanwhile, the inhibition of miR-143 potentiated intracellular Bcl-2 level
Zhou [30]	H_2_O_2_ → p53, miR-143 ┤ATG2B, Bcl-2, and LC3-I	The chemoresistance of OS tumor cells to doxorubicin is associated with the downregulation of miR-143 expression, activation of ALDH1^+^CD133^+^cells, activation of autophagy, and inhibition of cell death
Fang [31]	Negative correlation between COX-2 and miRNA-143	COX-2 expression in the tumor tissue and blood samples of patients with OS increases significantly along with the degree of tumor malignancy, and this is accompanied by a decreased expression of miRNA-143; A negative correlation between COX-2 and miRNA-143 may exist in the progression of OS
Shimbo [3]	–	Exosome-formed miR-143 was easily transferred into recipient cells and suppressed the migration of the 143B OS cell line
Li [32]	TGF-β1(Smad 2/3 pathway)┤miR-143	TGF-β1 suppressed miR-143 expression through a Smad 2/3-dependent pathway
Wang [33]	miR-143┤EGFR → ERK/MAPK → MMP9	MiR-143 inhibits EGFR signaling through its downstream ERK/MAPK signaling cascades to control MMP9 expression in OS. Thus, miR-143, EGFR, and MMP9 are therapeutic targets for inhibiting OS invasion
Ye [34]	Propofol → miR-143 ┤MMP-13	Propofol may have antitumor potential in OS, which is partly due to the downregulation of MMP-13 expression by miR-143
Li [35]	Notch-1┤miR-143	Diallyl trisulfide could be useful for inhibiting OS development and progression via suppression of Notch-1 signaling, accompanied by downregulation of Hes-1, VEGF, MMP-2 and MMP-9, as well as upregulation of specific tumor-suppressive miRNAs (miR34a, miR-143, miR-145 and miR-200b/c)
Ouyang [8]	–	The circulating levels of miR-143 were significantly decreased in patients with OS compared with controls
Hu [36]	–	The differential expression profiles of miRNAs between OS and osteoblast cell lines were investigated by miRNA microarrays and real-time quantitative PCR (RT-qPCR). A total of 268 miRNAs were identified that were significantly dysregulated in OS compared with the osteoblast cell line, including miR-9, miR-99, miR-195, miR-148a and miR-181a, which had been validated as overexpressed, and miR-143, miR-145, miR-335 and miR-539, which were confirmed to be downregulated
Osaki [37]	miR-143 ┤MMP-13	The downregulation of miR-143 correlates with the lung metastasis of human OS cells by promoting cellular invasion, probably via MMP-13 upregulation
Zhang [38]	miR-143┤Bcl-2	Bcl-2, an important antiapoptotic molecule, was identified to be a novel direct target of miR-143, and the proapoptotic function of miR-143 is further suggested to be mainly through the targeting of Bcl-2 expression

┤: inhibitory roles, → : stimulatory roles, *miR* MicroRNA, *Circ-RNA* circular RNA, *lncRNAs* long non-coding RNAs, *MSCs* stem cells, *MMP* matrix metalloproteinase, *PAI-1*: plasminogen activator inhibitor-1, KIAA1429:VIRMA, *COX-2* cyclooxygenase-2, *MAPK7* Mitogen activated protein kinase 7, *EGFR* Epidermal growth factor receptor, *FOSL2* FOS-Like antigen 2, *EVs* extracellular vesicles

**Table 2:** The information of others sarcoma

Author	disease	Genes/proteins affected	Functions
Kapodistrias [23]	Liposarcoma	–	miR-155, miR-21, miR-143, miR-145 and miR-451 that are implicated in liposarcoma, as novel formalin-fixed paraffin-embedded tissue biomarkers
Urdinez [22]	Chondrosarcoma	miR-143/145 ┤ FSCN1	miR 143/145/FSCN1 as important players in chondrosarcoma progression. Restoration of miR143/145 levels in tumors or direct FSCN1 targeting may hold potential as novel therapeutic approaches to chondrosarcoma
Ugras [20]	Liposarcoma	miR-143-3p ┤ BCL2, TOP2A, PRC1, and PLK1	Restoring miR-143 expression in dedifferentiated liposarcoma cells inhibited proliferation, induced apoptosis, and decreased expression of BCL2, TOP2A, PRC1, and PLK1. The downregulation of PRC1 and its docking partner PLK1 suggests that miR-143 inhibits cytokinesis in these cells. In support of this idea, treatment with a PLK1 inhibitor potently induced G2/M growth arrest and apoptosis in liposarcoma cells
De Vito [21]	Ewing Sarcoma	TARBP2	The miRNA profile of Ewing sarcoma family tumor cancer stem cells is the result of reversible disruption of TARBP2-dependent miRNA maturation. Restoration of TARBP2 activity and systemic delivery of synthetic forms of either of two of its targets, miRNA-143 or miRNA-145, inhibited Ewing sarcoma family tumor cancer stem cells clonogenicity and tumor growth in vivo

**Table 3:** The characteristics of the studies about mice

Studies	Characteristics of animals	Animal groups	OS xenograft methods	Experimental Groups (miR-143 overexpressed)	Control groups	Outcomes
Sun [7]	BALB/c nude mice (4 weeks)	6/6	Subcutaneous	143B + miR-143	143B + NC	Tumor volume Tumor weight Mice survival number
Li [28]	male BALB/c nude mice (4–6 weeks)	4/4	Subcutaneous	U2-OS + miR-143	U2-OS + control	Tumor volume Tumor weight
Zhou [30]	Balb/C nude mice (about 20 g)	9/9/9/9	Subcutaneous	U2-OS + miR-143	U2-OS + NC U2-OS + Dox U2-OS + miR-143 + DOX	Tumor weight
Zhou [30]	Balb/C nude mice (about 20 g)	9/9/9/9	Subcutaneous	SAOS-2 + miR-143	SAOS-2 + NC SAOS-2 + Dox SAOS-2 + miR-143 + DOX	Tumor weight
Osaki [37]	athymic mice	10/10	Intratibial	143B + miR-143	143B + NC	Tumor weight Mice survival number
Zhang [38]	female BALB/c athymic nude mice (4 weeks)	6/6	Subcutaneous	MG63 + miR-143	MG63 + NC	Tumor volume
Zhang [38]	female BALB/c athymic nude mice (4 weeks)	6/6	Subcutaneous	U2-OS + miR-143	U2-OS + NC	Tumor volume

**Table 4:** The characteristics of the studies about human

References	Country	Number	Gender(F/M)	Age (H /L)	Anatomical site(H /L)	Outcomes
Zhao [25]	China	High level: 58 Low level: 36	High level:27/31 Low level: 17/19	28 patients ≤ 20 years, 34 patients > 20 years/ 13 patients ≤ 20 years, 19 patients > 20 years	22 in femur, 28 in tibia, 9 in others/13 in femur, 21 in tibia, 5 in others	Tumor size, Tumor size, Metastasis
Sun [7]	China	High level: 10 Low level: 23	High level: 4/6 Low level: 12/11	7 patients ≤ 20 years, 3 patients > 20 years/ 15 patients ≤ 20 years, 7 patients > 20 years	5 in femur, 2 in tibia,2 in humerus,1 in others/17 in femur, 10 in tibia, 4 in humerus,2 in others	Tumor size, Tumor grade, Survival curve, expression level of miR-143
Dong [15]	China	High level: 19 Low level: 9	High level: 11/8 Low level: 3/6	11 patients < 18 years, 8 patients ≥ 18 years / 4 patients < 18 years, 5 patients ≥ 18 years	NR	Tumor size, Tumor grade, Metastasis, Recurrence
Hirahata [4]	Japan	High level: 3 Low level: 19	NR	NR	NR	Metastasis
Liu [29]	China	A total of 5 patients	NR	NR	NR	Expression level of miR-143
Zhou [30]	China	High level:20 Low level: 25	NR	NR	NR	Survival curve, Expression level of miR-143
Chen [40]	China	No metastasis: 35 Metastasis: 31	High level: 13 Low level: 15	NR	NR	Expression level of miR-143
Wang [33]	China	A total of 18 patients	NR	NR	NR	Expression level of miR-143
Wen [1]	China	20 cisplatin-sensitive and 20 cisplatin-resistant patients	NR	NR	NR	Expression level of miR-143
Li [28]	China	25 pair tissues	NR	NR	NR	Expression level of miR-143
Osaki [37]	Japan	No metastasis: 15 Lung metastasis: 7	NR	NR	NR	Expression level of miR-143, Metastasis

*NR* no report

### Quality of the included studies

In terms of Downs and Blacks score, all included studies were over 15. In NOS scale, 9 of 10 non- non-Randomized Controlled Trials (RCTs) had scored ≥ 6*. The lowest score was 5 *due to low quality of comparability and outcome. The quality of human studies was shown in Table 5. In CAMARADES criteria, the score of the study quality ranged from 5 to 7 out of a total 10 points, the outcome was illustrated in Table 6. In SYRCLE criteria, the score of the study quality ranged from 3 to 7 out of a total 10 points, the outcome was illustrated in Table 7. No study was identified with problems that could result in high risk of bias.

**Table 5:** The Downs and Blacks score and NOS scale

References	Country	Type	Study Quality					
			Downs and Black Score	NOS Scale				
				Selection	Comparability	Expose	Outcome	Total score
Wen [1]	China	Cohort studies	20	****	*	–	**	*******
Zhao [25]	China	Cohort studies	21	***	**	–	**	*******
Sun [7]	China	Cohort studies	23	***	**	–	**	*******
Dong [15]	China	Cohort studies	23	**	**	–	**	******
Hirahata [4]	Japan	Cohort studies	20	***	**	–	**	*******
Li [28]	China	Cohort studies	19	***	**	–	*	******
Liu [29]	China	Cohort studies	16	***	*	–	*	*****
Zhou [30]	China	Cohort studies	20	**	**	–	**	******
Chen [40]	China	Cohort studies	21	***	**	–	**	*******
Wang [33]	China	Cohort studies	18	***	**	–	**	*******
Osaki [37]	Japan	Cohort studies	18	**	**	–	**	******

**Table 6:** The CAMARADES criteria

Publication	1	2	3	4	5	6	7	8	9	10	Score
Sun [7]	√	√	√				√	√	√	√	7
Li [28]	√	√	√				√	√		√	6
Zhou [30]	√	√	√				√	√		√	6
Osaki [37]	√		√					√	√	√	5
Zhang [38]	√	√	√				√	√		√	6

Studies fulfilling the criteria of (1) peer reviewed publication; (2) control of temperature; (3) random allocation to treatment or control; (4) blinded induction of ischemia; (5) blinded assessment of outcome; (6) use of anaesthetic without significant intrinsic neuroprotective activity; (7) animal model (aged, diabetic or hypertensive); (8) sample size calculation; (9) compliance with animal welfare regulations; and (10) statement of potential conflict of interests

**Table 7:** The SYRCLE criteria

Publication	1	2	3	4	5	6	7	8	9	10	Score
Sun [7]	Y	Y	Y	Y	U	Y	N	U	Y	Y	7
Li [28]	U	Y	U	U	U	Y	N	N	N	Y	3
Zhou [30]	Y	Y	Y	Y	U	Y	U	U	Y	Y	7
Osaki [37]	U	Y	Y	U	U	Y	N	N	U	Y	4
Zhang [38]	U	Y	U	Y	U	Y	N	U	Y	Y	5

(1) Were participants allocated randomly to experimental and control groups? If so, was this sequence adequately generated and applied? (2) Were the groups similar at baseline or were they adjusted for confounders in the analysis? The baseline characteristics considered to be important were the age of animal, sex of animal and housing arrangements. (3) Was the allocation adequately concealed? (4) Were the animals randomly housed during the experiment? (5) Were the caregivers and/or investigators blinded from knowledge of which intervention each animal received during the experiment? This is also known as allocation concealment. (6) Were animals selected at random for outcome assessment? In other words, were control animals and experimental animals recorded in groups? (7) Was the outcome assessor blinded? This could be either during analysis or data collection. (8) Were incomplete outcome data adequately addressed? (9) Are reports of the study free of selective outcome reporting? (10) Was the study apparently free of other problems that could result in high risk of bias?

### Mice studies

#### Tumor volume

1. Final tumor volume

**Fig. 3:**
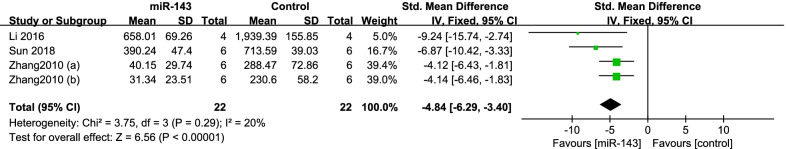
Forest plot diagram showed the standard mean difference in tumor volume

Three studies [7, 28, 38] compared the tumor volume. The data of them were pooled to do analysis. There was no significant heterogeneity between the studies (*P* = 0.29; *I*^2^ = 20%); therefore, the fixed-effects model was used. It showed the miR-143 up-regulation group had significantly smaller tumor volume (SMD = − 4.84, 95% CI: − 6.29 to − 3.40, *P* < 0.001; Fig. 3).

(2) subgroup analysis by follow-up period

**Fig. 4:**
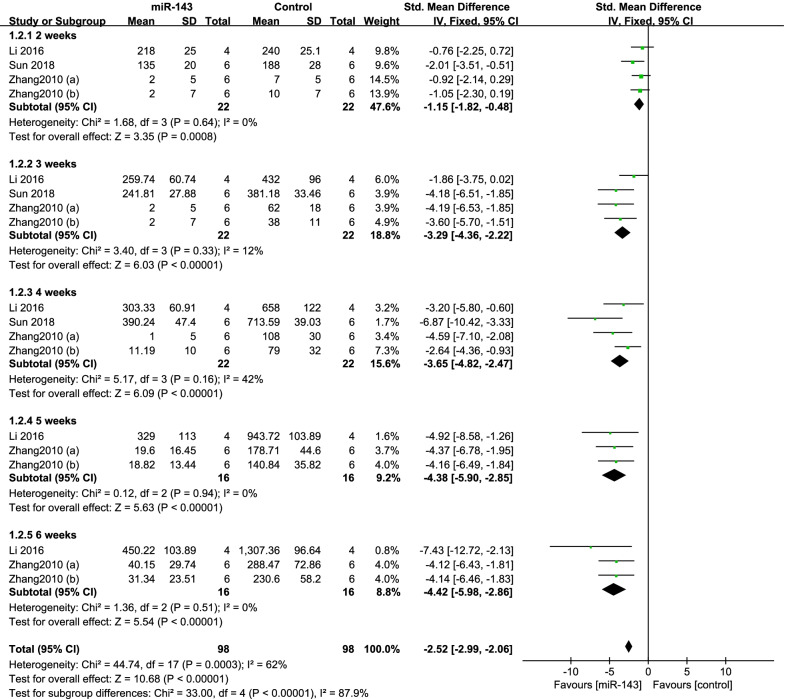
Forest plot diagram showed the standard mean difference in tumor volume with subgroup analysis by follow-up period

According to the follow-up period subgroup analysis was performed. We divided the follow-up period into five time periods: 2 weeks (*P* = 0.64; *I*^2^ = 0%), 3 weeks (*P* = 0.33; *I*^2^ = 12%), 4 weeks (*P* = 0.16; *I*^2^ = 42%), 5 weeks (*P* = 0.94; *I*^2^ = 0%) and 6 weeks (*P* = 0.51; *I*^2^ = 0%), so the fixed-effects model was performed. The pooled results manifested that the miR-143 up-regulation group had significantly better outcomes in the group of 2 weeks (SMD = − 1.15, 95% CI: − 1.82 to − 0.48, *P* = 0.0008; Fig. 3), 3 weeks (SMD = − 3.29, 95% CI: − 4.36 to − 2.22, *P* < 0.001; Fig. 3), 4 weeks (SMD = − 3.65, 95% CI: − 4.82 to − 2.47, *P* < 0.001; Fig. 3), 5 weeks (SMD = − 4.38, 95% CI:− 5.90 to − 2.85, *P* < 0.001; Fig. 3) and 6 weeks (SMD = − 4.42, 95% CI: − 5.98 to − 2.86, *P* < 0.001; Fig. 4).

(3) subgroup analysis by type of cell

**Fig. 5:**
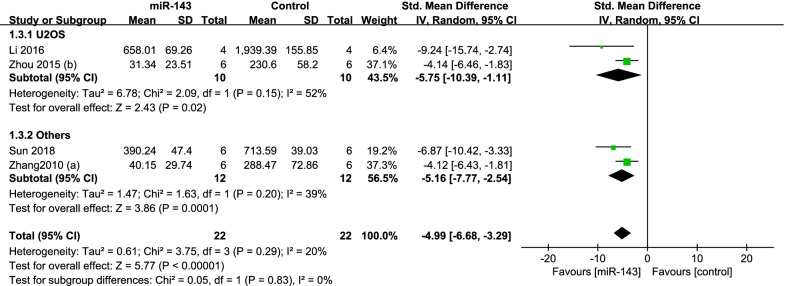
Forest plot diagram showed the standard mean difference in tumor volume with subgroup analysis by type of cell

According to the type of cell, subgroup analysis was performed. We divided the studies in two group: U2OS (*P* = 0.15; *I*^2^ = 52%) and others (MG63 and 143B) (*P* = 0.20; *I*^2^ = 39%), so the Random-effects model was performed. The pooled results manifested that the miR-143 up-regulation group had significantly better outcomes in the group of U2OS (SMD = − 5.75, 95% CI: − 10.39 to − 1.11, *P* = 0.02; Fig. 4) and others (SMD = − 5.16, 95% CI: − 7.77 to − 2.54, *P* = 0.0001; Fig. 5).

#### Tumor weight

1. Final tumor weight

**Fig. 6:**
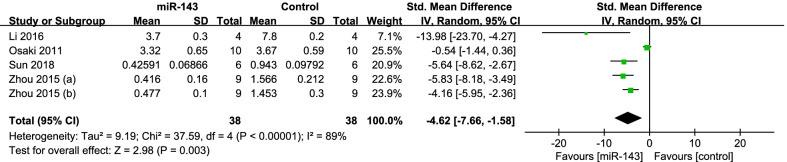
Forest plot diagram showed the standard mean difference in tumor weight

Four studies [7, 28, 30, 37] compared the tumor weight. The data of them were pooled to do analysis. There was significant heterogeneity between the studies (*P* < 0.001; *I*^2^ = 89%); therefore, the random-effects model was used. It showed the miR-143 group had significantly lighter tumor weight (SMD = − 4.62, 95% CI: − 7.66 to − 1.58, *P* = 0.003; Fig. 6).

(2) subgroup analysis by type of cell

**Fig. 7:**
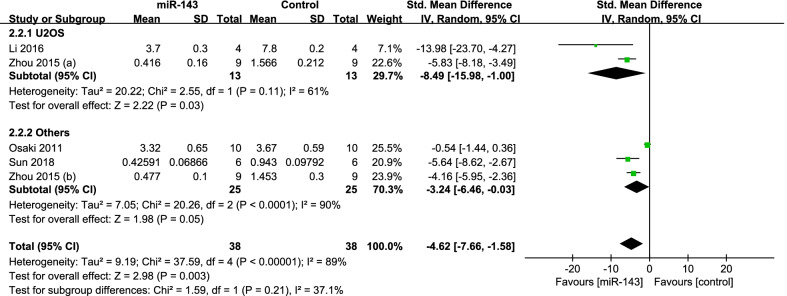
Forest plot diagram showed the standard mean difference in tumor weight with subgroup analysis by type of cell

According to the type of cell, subgroup analysis was performed. We divided the studies into two group: U2OS (*P* = 0.11; *I*^2^ = 61%) and others (143B and Saos-2) (*P* < 0.001; *I*^2^ = 90%), so the random-effects model was performed. The pooled results manifested that the miR-143 group had better outcomes in the group of U2OS (SMD = − 8.49, 95% CI: − 15.98 to − 1.00, *P* = 0.03; Fig. 6) and others (SMD = − 3.24, 95% CI: − 6.46 to − 0.03, *P* = 0.05; Fig. 7).

#### Survival rate of mice

**Fig. 8:**
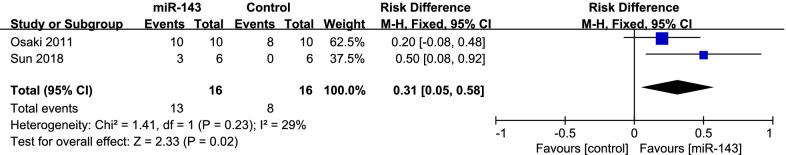
Forest plot diagram showed the risk difference in survival rate of mice

Two studies [7, 37] reported survival rate of mice. There were 13 of 16 (81.25%) mice in the miR-143 group and 8 of 16 (50%) patients in the low-level group. No significant heterogeneity was detected (*P* = 0.23; *I*^2^ = 29%); therefore, the fixed-effects model was used. The final outcomes manifested that the high-level of miR-143 had a significantly better outcomes in survival rate of mice (RD = 0.31, 95% CI: 0.05–0.58, *P* = 0.02; Fig. 8).

### Human studies

#### Survival rate

**Fig. 9:**
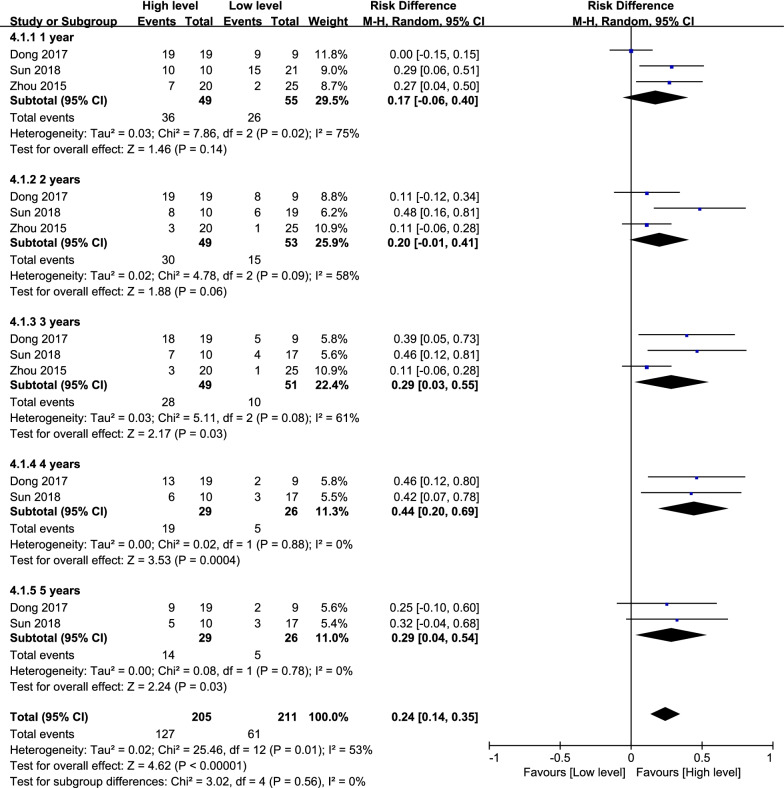
Forest plot diagram showed the risk difference in survival rate of human

The survival rate was reported in 3 studies [7, 15, 30]. The data of them were pooled to do analysis. According to the follow-up period, subgroup analysis was performed using a random effects model, we divided the follow-up period into five time periods:1 year (*P* = 0.02; *I*^2^ = 75%), 2 years (*P* = 0.09; *I*^2^ = 58%), 3 years (*P* = 0.08; *I*^2^ = 61%), 4 years (*P* = 0.88; *I*^2^ = 0%) and 5 years (*P* = 0.78; *I*^2^ = 0%). The pooled results manifested that the high-level group had significantly better outcomes in the group of 3 years (RD = 0.29, 95% CI: 0.03 to 0.55, *P* = 0.03; Fig. 8), 4 years (RD = 0.44, 95% CI: 0.20 to 0.69, *P* = 0.0004; Fig. 8) and 5 years (RD = 0.29, 95% CI: 0.04 to 0.54, *P* = 0.03; Fig. 8). The pooled results manifested no significant difference between the two groups in 1 year (RD = 0.17, 95% CI: − 0.06 to 0.40, *P* = 0.14; Fig. 8) and 2 years (RD = 0.20, 95% CI: − 0.01 to 0.41, *P* = 0.06; Fig. 9).

#### Lung metastasis

**Fig. 10:**
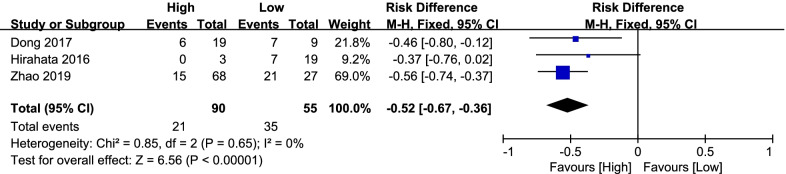
Forest plot diagram showed the risk difference in lung metastasis

Three studies [4, 15, 25] reported lung metastasis. There were 21 of 90 (23.33%) patients in the high-level group and 35 of 55 (63.64%) patients in the low-level group. No significant heterogeneity was detected (*P* = 0.65; *I*^2^ = 0%); therefore, the fixed-effects model was used. The final outcomes manifested that the high-level of miR-143 had a significantly better outcomes in lung metastasis (RD = − 0.52, 95% CI: − 0.67 to − 0.36, *P* < 0.001; Fig. 10).

#### Tumor grade

**Fig. 11:**
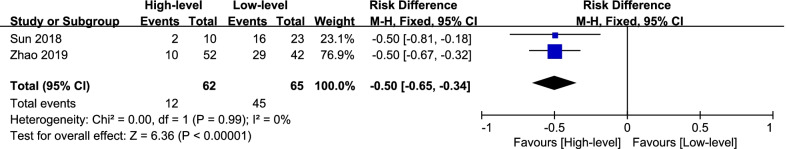
Forest plot diagram showed the risk difference in low tumor grade

Two studies [7, 25] reported tumor grade. In these studies, 12 of 62 (19.35%) patients had low tumor grade in the high-level group and 45 of 65 (69.23%) patients in the low-level group. No significant heterogeneity was detected (*P* = 0.99; *I*^2^ = 0%); therefore, the fixed-effects model was used. The final outcomes manifested that the high-level of miR-143 had a significantly better in the outcomes of low tumor grade (RD = − 0.50, 95% CI: − 0.65 to − 0.34, *P* < 0.001; Fig. 11).

#### Expression of miR-143

1. Comparisons between the human OS tissue and the adjacent normal tissue

**Fig. 12:**
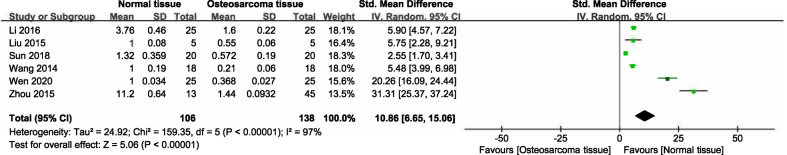
Forest plot diagram showed the standard mean difference in expression of miR-143 compared between the OS tissue and the adjacent normal tissue

Six studies [1, 7, 28–30, 33] compared the expression of miR-143 between the OS tissue and the adjacent normal tissue. The data of them were pooled to do analysis. Significant heterogeneity was found between the studies (*P* < 0.001; *I*^2^ = 97%); therefore, the random-effects model was used. The results showed that miR-143 was significantly down-regulated in human OS tissue. (SMD = 10.86, 95% CI: 6.65 to 15.06, *P* < 0.001; Fig. 12).

2) Comparisons between the human OS tissue with metastasis and OS tissue without metastasis

**Fig. 13:**
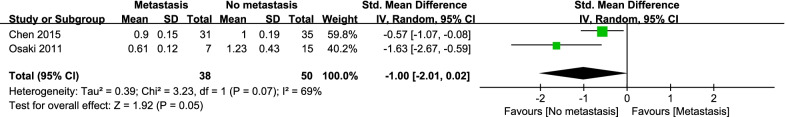
Forest plot diagram showed the standard mean difference in expression of miR-143 compared between the metastasis tissue and no metastasis tissue

Two studies [37, 40] compared the OS tissue expression of miR-143 between the metastasis and no metastasis. The data of them were pooled to do analysis. There was no significant heterogeneity between the studies (*P* = 0.07; *I*^2^ = 69%); therefore, the fixed-effects model was used. It showed miR-143 was down-regulated in human OS tissue with metastasis (SMD = − 1.00, 95% CI: − 2.01 to 0.02, *P* = 0.05; Fig. 13).

### Sensitivity analyses

One study was individual deleted each time to observe its influence on the pooled SMD or RD. The results showed that no study could substantially affect the pooled SMD or RD in the present meta-analysis.

### Database analysis

The data-set GSE65071 based on the platform of GPL19631 and GSE28423 based on GPL8227 were chosen for analysis. We found that miR-143 is significantly down regulated in plasma samples from patients with osteosarcoma (log_2_FC = − 2.13; *P* < 0.05) and in osteosarcoma cell lines (log_2_FC = − 3.28; *P* < 0.05).

## Discussion

The results of our meta-analysis demonstrated that the upregulation of miRNA-143 expression could significantly decrease the mice tumor volume, reduce mice tumor weight, improve mice survival rate. In addition, high expression of miR-143 significantly could improve human survival rate, decrease the risk of lung metastasis, and reduce the incidence of the high-grade OS. In the expression level of miR-143, the OS tissue was significantly lower than the adjacent normal tissue, and the patient without metastasis was significantly higher than the patient with metastasis.

Tumor volume and tumor weight are two main indicators in the animal experiment. Our study found that by restoring the normal expression of miR-143 in OS mice model, the volume and weight of tumor could be significantly reduced. Han et al. found that miR-143-3p could decrease KIAA1429 expression which could significantly promote the OS progression in the mice [14]. Zhang et al. found the overexpressed of circUBAP2 promoted mice OS growth by inhibiting the expression of miR-143 [26]. In addition, we also found that the survival rate of mice with high miR-143 expression was significantly higher than that of mice with low miR-143 expression. Osaki et al. found miRNA-143 regulated mice OS metastasis by regulating Matrix Metalloprotease-13 (MMP-13) [37]. Yu et al. reported that long non-coding RNA TUG1 knockdown promoted tumorigenesis, peritoneal spread, and metastasis of mice OS and by mediating HIF-1α via miR-143-5p [5]. Summarize the evidence, miR-143 upregulation can inhibit the growth of OS in mice and improve the survival rate of mice.

In the results of human, we found that the human survival rate of miR-143 high expression group was significantly higher than that of miR-143 low expression group from the third year of follow-up. The high expression group had a significantly higher probability of low-grade OS. In addition, we also found that the expression of miR-143 in tumor tissue was significantly lower than that in surrounding normal tissues, and the expression level of miR-143 in metastatic primary OS tissue was significantly lower than that of patients without metastasis. Zhang reported miR-143 is down-regulated in primary osteosarcoma [38]. Fang found that the miRNA-143 level in the tumor tissue declined related to disease severity in OS patients [31]. Yu reported that survival and recurrence-free survival of OS patients with relatively high expression of lncRNA TUG1, which could inhibit the expression of miR-143, was significantly lower than the low expression [5]. In Zhang’s study, Low expression of circular RNA UBAP2 had a significantly better survival rate than high expression which inhibited the expression of miR-143[26]. By analyzing plasma between OS patients and healthy people, Ouyang et al. reported miR-143 significantly decreased in OS patients compared with controls. In addition, level of miR-143 significantly decreased in patients with metastasis compared with no metastasis patients [8]. Zhang et al. reported that miR-143 expression is inhibited by lncRNA FOXD2-AS1 in drug-resistant cell lines, and the knockdown of LncRNA FOXD2-AS1 inhibits the resistance of human OS cells to cisplatin, promotes cell apoptosis and suppresses cell invasion and migration abilities [41]. Li et al. noted that bone marrow-derived mesenchymal stem cells-derived extracellular vesicles promote proliferation, invasion and migration of osteosarcoma cells via the MALAT1/miR-143/NRSN2/Wnt/β-catenin axis [42]. Bi et al. reported the knockdown of lncRNA colon cancer‑associated transcript 2 could inhibit the proliferation and metastasis of OS cells via targeting miR‑143[43]. Yang et al. pointed that miR-143 expression were significantly down-regulated in the serum from OS patients, and the OS patients with lower serum miR-143 expressions survived shorter than those with higher serum miR-143 expressions (*P* = 0.0421). They concluded that serum miR-143 may function as diagnostic and prognostic markers for OS [44]. Summarize the evidence, we concluded that miR-143 upregulation could inhibit the growth and invasion of OS in human and improve the survival rate.

In many other types of tumors such as esophageal squamous cell carcinoma, breast cancer, colon cancer, prostate cancer, and ovarian carcinoma, many researches confirm miR-143 is a tumor suppressor [15]. In addition, some studies have found that miR-143 can also inhibit tumor growth and invasion in other sarcomas. Urdinez et al. reported that miR-143 could decrease expression of BCL2, TOP2A, PRC1, and PLK1 to inhibit liposarcoma cell proliferation and induce liposarcoma cell apoptosis [22]. De Vito et al. reported miRNA-143 inhibited Ewing sarcoma family tumor cancer stem cell clonogenicity and tumor growth by targeting TARBP2 [18].

Recent studies have shown that noncoding RNAs also plays an important role in musculoskeletal diseases. For example, specific miRNA can regulate the expression of cytokines and coordinate the proliferation and differentiation of stromal cell lines involved in the composition of extracellular matrix [45], and siRNA also can be used to study the repair process of tendon and identify possible therapeutic targets in tendon healing [46]. In addition, miRNA may be involved in the diagnosis and treatment of osteoarthritis [47]. The main limitation of our research is that some results were related to the high heterogeneity of animal experiments. But the heterogeneity was acceptable due to different researchers, different laboratories, different techniques, and small size. In addition, for the results of high heterogeneity, we used a random-effects model. Another factor is the lack of relevant literature, which may affect the reliability of the results.

## Conclusions

We found that miR-143 could inhibit OS growth, prevent tumor metastasis and improve survival rate by analyzing the results of studies about mice and patients. Therefore, miR-143 may have potentially great value as a treatment and prognostic biological marker for OS. This will provide strong evidence for the development of animal experiments and clinical treatment of OS in the future. However, before converting miR-143 based treatments from animal research to human applications, more reliable animal and clinical trials are needed.

## Data Availability

All data are fully available without restriction.

## Abbreviations

OS: Osteosarcoma
miR-143: MicroRNA-143
CNKI: China national knowledge infrastructure
PMC: PubMed Central
PRISMA: Preferred reporting items for systematic reviews and meta-analysis
NOS: Newcastle–Ottawa Scale
SMD: Standard mean difference
CI: Confidence interval
RD: Risk difference
RCTs: Randomized controlled trials
FC: Fold change
